# Gray-scale and color duplex Doppler ultrasound of hand joints in the evaluation of disease activity and treatment in rheumatoid arthritis

**DOI:** 10.3325/cmj.2015.56.280

**Published:** 2015-06

**Authors:** Gordana Ivanac, Jadranka Morović-Vergles, Boris Brkljačić

**Affiliations:** 1 Department of Diagnostic and Interventional Radiology, Ultrasound Unit, University Hospital “Dubrava,” University of Zagreb School of Medicine, Zagreb, Croatia; 2 Department of Internal Medicine, Division of Clinical Immunology and Rheumatology, University Hospital “Dubrava,” University of Zagreb School of Medicine, Zagreb, Croatia; Ivanac et al: Ultrasound of hand joints in evaluation of rheumatoid arthritis

## Abstract

**Aim:**

To evaluate the role of gray-scale and color duplex-Doppler ultrasound (CDUS) in diagnosis of changes of hand joints and assessment of treatment efficacy in patients with rheumatoid arthritis (RA) by comparing qualitative and quantitative US parameters with clinical and laboratory indicators of disease activity.

**Methods:**

Ulnocarpal (UC), metacarpophalangeal (MCP), and proximal interphalangeal (PIP) joints in 30 patients with RA were examined by gray-scale and CDUS before and after six months of treatment. Morphologic and quantitative Doppler findings (synovial thickness, effusion quantity, vascularization degree, resistance index, velocities) were compared with clinical indicators of disease progression: disease activity score (DAS 28), Health Assessment Questionnaire (HAQ), rheumatoid factor (RF), erythrocyte sedimentation rate (ESR), and C reactive protein (CRP).

**Results:**

Clinical indicators changed significantly after treatment: ESR from 38.1 ± 22.4 mm/h to 27.8 ± 20.9 mm/h (*P* = 0.013), DAS 28 from 5.47 ± 1.56 to 3.87 ± 1.65 (*P* < 0.001), and HAQ from 1.26 ± 0.66 to 0.92 ± 0.74 (*P* = 0.030), indicating therapeutic effectiveness. In all MCP and UC joints we observed a significant change in at least one US parameter, in 6 out of 12 joints we observed a significant change in ≥2 parameters, and in 2 UC joints we observed significant changes in ≥3 parameters. The new finding was that the cut-off values of resistance index of 0.40 at baseline and of 0.55 after the treatment indicated the presence of active disease and the efficacy of treatment, respectively; also it was noticed that PIP joints can be omitted from examination protocol.

**Conclusion:**

Gray scale and CDUS are useful in diagnosis of changes in UC and MCP joints of patients with RA and in monitoring the treatment efficacy.

Rheumatoid arthritis (RA) is a chronic systemic inflammatory disease of unknown etiology, which primarily affects the synovia; persisting synovitis of symmetric peripheral joints can lead to structural damage of cartilage, bones, tendons, and ligaments (1). The standard modality used to estimate joint destruction in patients with RA is conventional radiography (CR), although its sensitivity to detect early erosions is relatively low and it cannot demonstrate the inflamed synovia (2-4). The classic radiographic signs of RA are osteoporosis, bone erosions, and joint space narrowing. The presence of bone erosions has considerable implications for the treatment and prognosis (3). For the correct diagnosis of RA it is necessary to analyze the changes on joints of hands and feet, and radiographic follow up of early polyarthritis is the integral part of patients’ treatment. Regression of bone erosions seldom occurs in patients who receive treatment and cannot be used as an indicator of treatment efficacy, and CR is not an adequate method to evaluate treatment success (2,4).

Early changes can be revealed much better by scintigraphy and magnetic resonance imaging (MRI); contrast-enhanced MRI enables early detection of inflammatory changes, as contrast is needed to differentiate fluid from panus. MRI is also useful to estimate the treatment success (4,5). In a recent systematic review, the diagnostic value of MRI for RA varied widely across studies, and in many studies MRI definitions and scoring methods were not standardized (5). There are very few studies comparing prognostic and monitoring role of ultrasound (US) and MRI in RA (6).

Several studies confirmed the usefulness of US for the detection of joint changes in RA and patients follow-up during therapy, as well as for the guidance for intra-articular procedures (7). In a national survey of Spanish rheumatologists, the utility of US in the routine clinical practice was very high (7.8 at scale 0-10) in rheumatology in general, but particularly for the diagnosis and treatment decision making in RA (8). American College of Rheumatology recently published a report on the reasonable use of US in rheumatology clinical practice (9). According to ACR/EULAR criteria for the classification of RA, US examination is a valid method for detection of active synovitis (10) and it was even shown that US could detect activity in patients with clinical remission (11).

Gray-scale US enables accurate visualization of inflamed synovia, joint fluid, and paraarticular changes. Color and power Doppler are excellent for visualization of blood flow in inflamed joints, so that the stage of inflammatory changes can be estimated and the efficacy of treatment evaluated by analyzing the decrease in synovial thickening and reduction of vascularization (7-10,12). In patients with incipient RA changes, more erosions were diagnosed with US than with CR, and US provided a good estimate of the progression of early disease (13).

The aim of this study was to evaluate the role of gray-scale and color duplex Doppler ultrasound (CDUS) in diagnosis and follow-up of patients with RA during at least six-months of treatment by comparing qualitative and quantitative US parameters to several standard clinical indicators of the disease activity. The study design differed from previous studies because cut-off values of resistance index (RI), which might indicate the presence of active disease, were determined before and after the treatment. An additional aim was to examine which hand joints should be examined and which could be omitted from the examination, thus reducing examination time. Finally, the examination performed in a highly-standardized fashion by a single, experienced operator, using the top-quality scanner represented a rather exceptional setting for clinical studies about the utilization of CDUS in RA.

## Materials and methods

This prospective study consecutively included 30 patients with RA in whom adequate US exam could be performed on all joints (21 women and 9 men, age median 53 years, range 17-79) during one-month period in 2010. The patients with large deformities in whom US could not be properly performed were excluded. US examinations were performed at the baseline and after six months of treatment on the day of clinical examination and disease activity score was determined. All patients were diagnosed and treated at the Department of Clinical Immunology and Rheumatology of University Hospital “Dubrava,” Zagreb, Croatia, and were recruited during routine diagnostic procedures. CR of both hands of each patient was performed to determine the stage of the disease and the presence of erosions, subluxations, or luxations that would prevent the technical performance of the complete US exam. Gray-scale and CDUS were used to evaluate morphologic changes of inflamed joints, assess the degree of their vascularization, quantify spectral changes, and assess effects of treatment. US findings were compared to clinical findings and laboratory inflammatory parameters.

Examinations were performed on ulnocarpal (UC), metacarpophalangeal (MCP), and proximal interphalangeal (PIP) joints at the baseline and after at least six-months of treatment (range 184-200 days in all patients; the total length of the study was 230 days encompassing the first and the last examination in all patients). Twenty-two joints of both hands (10 MCP, 10 PIP, 2 UC) were examined in each patient.

Twenty eight patients were treated with metothrexate in dosage ranging 17.5-25 mg/week and 2 patients with Arava (leflunomid) 20 mg every day. Among 28 patients treated with metothrexate, 8 patients were treated with anti-TNF-alpha inhibitors. Three were treated with Humira (adalimumab) 40 mg every second week subcutaneously, and 5 were treated with Enbrel (etanercept) 25 mg once a week subcutaneously. Twenty two patients who were treated with metothrexate or Arava, but not with biologics, were also treated with Salazopirin EN 500 (sulphasalazine) 2 g per day. Among the latter, 14 patients were additionally treated with glucocortcoids (metilprednisolone <10 mg/d). Medical treatment was performed using standard and established dosages according to the established clinical practice: all patients were treated by a single, experienced clinical rheumatologist and immunologist with over 20 years of experience (second author).

Rheumatoid factor (RF) and inflammatory parameters (erythrocyte sedimentation rate, ESR, and C-reactive protein, CRP) were measured at the baseline and after six months of treatment. The clinical Disease Activity Score – CRP in 28 joints (DAS 28), which is an indicator of the disease activity, was derived using the formula: DAS28 = 0.56 × √TJ+0.28 × √SJ+0.36 × ln(CRP+1)+ 0.014 × GH^+^0.96, where TJ is the number of tender joints (0-28); SJ the number of swollen joints (0-28); GH general health scale or patients global assessment of disease activity (patients themselves estimate their condition on a 1-100 scale); and CRP C-reactive protein (14,15). Tender and swollen joint counts were determined in 28 joints that include shoulders, elbows, knees, wrists, MCP I-V joints, and PIP I-V joints. DAS 28 values between 2.6 and 3.2 indicated low disease activity, between 3.2-5.1 moderate disease activity, and higher than 5.1 high disease activity. DAS 28 values below 2.6 indicated remission.

Every patient completed the Health Assessment Questionnaire (HAQ), a self-administered standardized questionnaire about the individual daily functioning abilities (14,16). The questionnaire consists of 43 questions about the patient's daily life functioning performance alone or with the help from another person concerning clothing, hygiene, standing, nutrition, walking, reaching for items, receiving items, opening of closed items, and small household chores. The questionnaire was translated to Croatian and previously validated. The answers are the following: without any difficulties – 0, difficult – 1, very difficult – 2, impossible – 3. The total final HAQ score is expressed as a mean value that is derived from the eight separate scores. The questionnaire also contains visual-analog scale for the estimation of pain and complete status (14,16).

US examinations of 22 UC, MCP, and PIP joints per patient were performed in a highly standardized fashion, using the high-performance ultrasound scanner Logiq 9 (General Electric Medical Systems, Milwaukee, WI, USA), with the high frequency matrix probe (14 MHz). All examinations were performed by the first author, with fifteen years’ experience in the field of musculoskeletal US, on the same scanner with the same presets for each patient; the interobserver variability was not the issue in this study. All joints were examined from palmar and extensor sides. The right hand was examined first. Gray-scale was performed using the “compound mode” and “native harmonic” in standard dynamic interval with B-gain set at 2/3 of the maximum size. The presence of effusion, thickening of synovia, and joint erosions was estimated.

Findings were categorized semiquantitatively into four groups, according to Szkudlarek et al (17). The presence of joint effusion was categorized as: 0 – no fluid; 1 – minimal fluid; 2 – moderate fluid; 3 – large quantity of fluid; the synovial thickening as: 0 – no synovial thickening; 1 – minimal synovial thickening; 2 – synovial thickening that exceeds the line connecting periarticular bones, without extension to the bone dyaphisis; 3 – synovial thickening that exceeds the line which connects periarticular bones and also extends on dyaphisis; and bone changes as: 0 – regular bone surface, 1 – irregular bone surface, 2 – small defect on bone surface, 3 – defect on bone surface with destruction.

**Figure 1:**
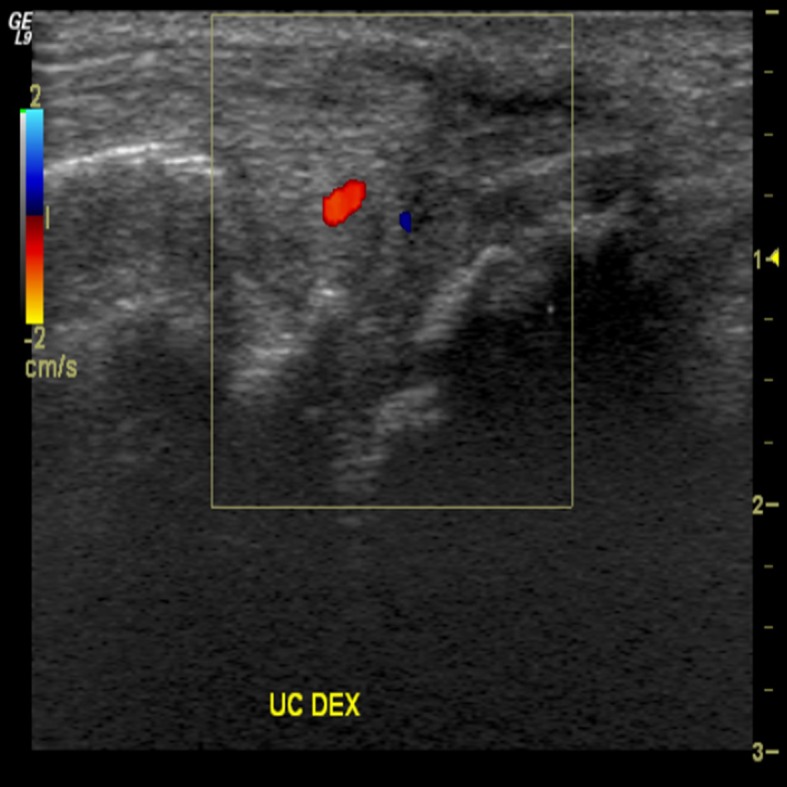
Vascularization in ulnocarpal joint – group 1: Doppler color signal in only one blood vessel.

**Figure 2:**
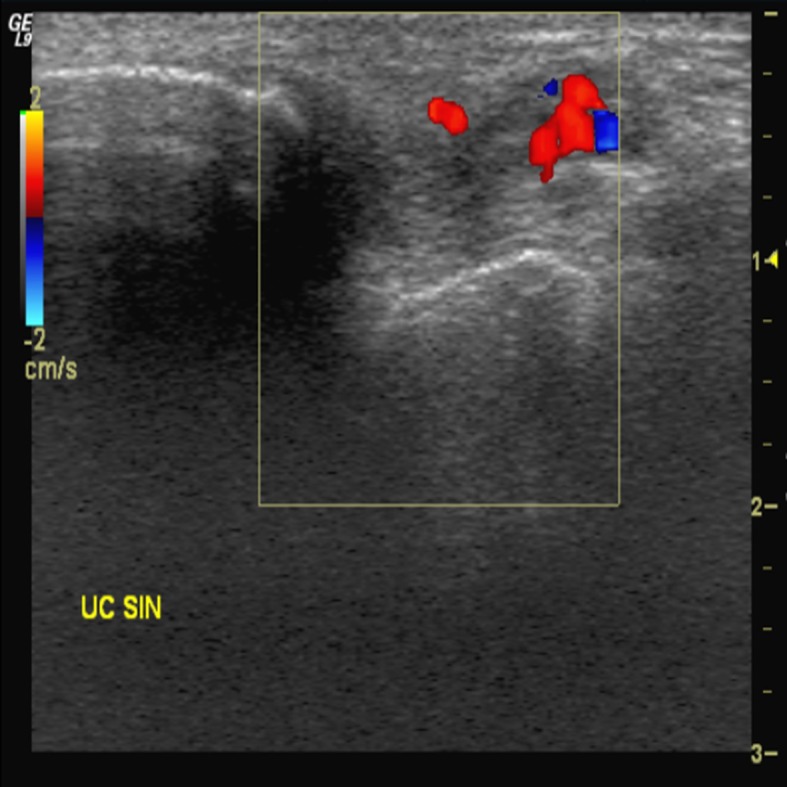
Vascularization in ulnocarpal joint – group 2: more Doppler signals in vessels, but on the surface that is smaller than the half of the complete size of synovia.

**Figure 3:**
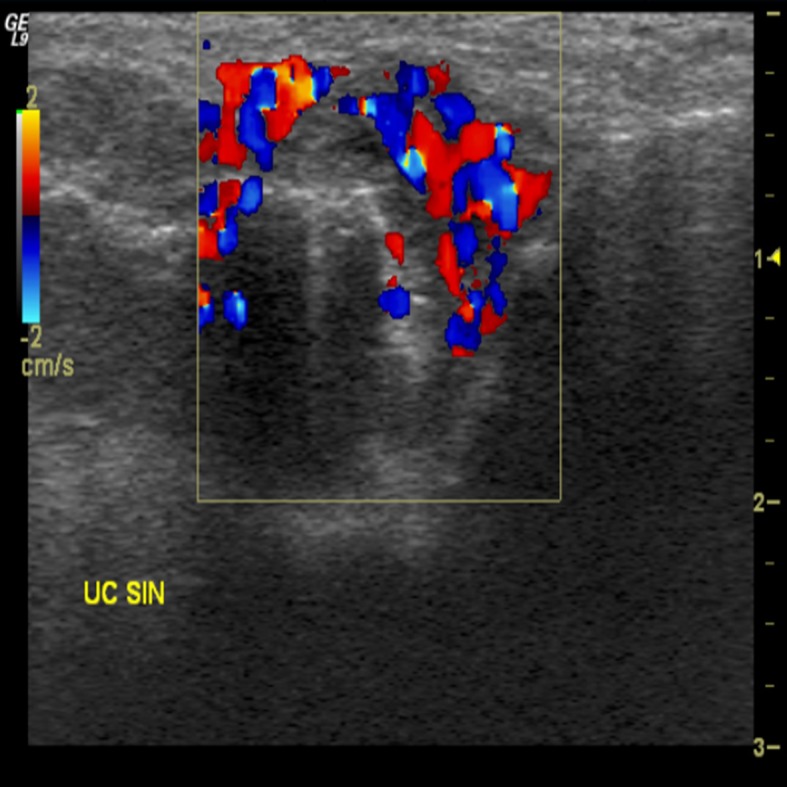
Vascularization in ulnocarpal joint – group 3: more Doppler signals in vessels on the surface larger than the half of the synovial size.

Color Doppler (CD) was used to estimate the degree of vascularization of the inflamed synovia. Technical parameters were always the same to enable accurate comparison of the results. The lowest wall-filter that eliminated vessel wall noise was used, color gain was set at the 2/3 of the maximum value, the lowest pulse repetition frequency was used that does not cause aliasing, and the priority level was set at 90% so that the slow flow in small vessels could be visualized. Angle correction was performed carefully, as this is crucial for obtaining accurate values. The single color scale with red-orange-yellow shades was always used. CD findings were categorized into four groups: 0 – no vascularization, 1 – color signal in only one blood vessel, 2 – more color signals in vessels, but on the surface that is smaller than the half of the complete size of the synovia, 3 – more signals in vessels on the surface larger than the half of the synovial size (17) (Figures 1,2,3).

In the joints where vascularization was observed, the quantitative values were measured using the spectral frequency analysis of arterial flow: peak systolic velocity (PSV), end diastolic velocity (EDV), and RI. Altogether 1320 joints in 30 patients were examined by US, 660 joints at baseline and the same joints after six months of therapy.

Clinical and laboratory parameters – RF, CRP, ESR, DAS 28, and HAQ values – were determined on the day of the first US examination and again after at least six months of treatment, at the time when the patients were reexamined by US. Results were compared with the gray-scale findings and with the qualitative and quantitative CDUS parameters. The validity of diagnostic procedure was calculated for different cut-off values of RI that were arbitrarily determined (RI 0.40; RI 0.45; RI 0.50; RI 0.55) for the groups before and after therapy in relation to DAS28 score of >3.2 to determine the best cut-off values of RI indicating active disease before the treatment and successful treatment in the post-treatment group of patients. All patients signed informed consent and the research was approved by the Ethics Committees of the Institution (University Hospital) and of the University of Zagreb School of Medicine.

### Statistical analysis

The variables were analyzed using descriptive measures. Quantitative variables distribution was tested by Kolmogorov-Smirnov test. Variables with normal distribution were analyzed with paired samples *t* test and Pearson correlation analysis and variables with not-normal distribution with Wilcoxon’s test of equivalent pairs and Spearman’s correlation analysis. Qualitative variables and dependent samples were tested with χ˛-test, Stuart-Maxwell's test, and McNemar's test. Measures of diagnostic procedure validity were determined (sensitivity, specificity, positive predictive value, negative predictive value, and accuracy). Statistical analysis was performed using STATISTICA, ver. 10 (StatSoft Inc., Tulsa, OK, USA). The level of statistical significance was set at *P* < 0.05.

## Results

### Changes of laboratory parameters, DAS 28 and HAQ

ESR, DAS 28, and HAQ score significantly decreased after treatment compared to the baseline values (*P* = 0.013, *P* < 0.001, *P* = 0.030, respectively, *t* test for sample pairs); changes of HAQ and DAS 28 scores indicated successful treatment as the disease activity was reduced. RF values also decreased significantly, while CRP decreased but not significantly (*P* = 0.015 and *P* = 0.061, respectively, Wilcoxon’s test of equivalent pairs) (Table 1).

**Table 1:** Baseline and post-treatment levels of erythrocyte sedimentation rate (ESR), disease activity score (DAS 28), Health Assessment Questionnaire (HAQ), rheumatoid factor (RF), and C reactive protein (CRP)

	Baseline level	Post-treatment level/average	*P*
ESR (mm/h) (mean ± standard deviation)	38.1 ± 22.4	27.8 ± 20.9	0.013*
DAS28 (mean ± standard deviation)	5.47 ± 1.56	3.87 ± 1.65	<0.001*
HAQ (mean ± standard deviation	1.26 ± 0.66	0.92 ± 0.74	0.030*
RF (IU/mL) (median, range)	202.75 (2.1-1205.0)	104.25 (1.0-443.0)	0.015^†^
CRP (mg/L) (median, range)	31.65 (0.70-124.5)	22.8 (0.30-107.2)	0.061^†^

*t test for sample pairs.

†Wilcoxon’s test of equivalent pairs.

### Changes of qualitative sonographic parameters: erosions, synovial thickening, synovial effusions, and vascularization

Erosions, synovial thickening, and synovial effusions were evaluated by gray-scale US. The number and the degree of joint erosions did not change significantly between baseline and the end of treatment. Synovial thickening was significantly reduced in 7 out of 12 MCP and UC joints, synovial effusion in 2 MCP joints, and vascularization, estimated by CDUS, in one UC and one MCP joint (Table 2).

**Table 2:** Changes of qualitative ultrasonographic parameters of synovial thickening, effusions, and degree of vascularization, which were significant after treatment on different joints

Joint	*P* value (Stuart Maxwell-test)
Synovial thickness	
UC L	0.046
MCP 1 R	0.046
MCP 1 L	0.020
MCP 3 R	0.024
MCP 4 L	0.012
MCP 5 R	0.018
MCP 5 L	0.009
Effusions	
MCP 1 R	0.035
MCP 2 L	0.044
Vascularization degree	
UC R	0.002
MCP 3 R	0.024

*UC – ulnocarpal; MCP – metacarpophalangeal; R – right; L – left.

### Changes of quantitative Doppler parameters

**Figure 4:**
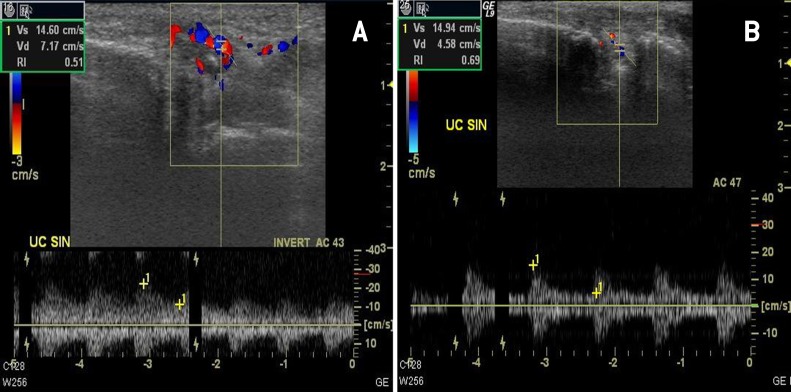
(**A**) Example of increased vascularization of the group 2 in the left ulnocarpal joint with spectrum indicated inflammation and low resistance index (RI) of 0.51. (**B**) The same left ulnocarpal joint after treatment demonstrated a decrease in vascularization and now can be classified as group 1, with RI value of 0.69

Quantitative spectral parameters of PSV, EDV, and RI were measured by CDUS. After treatment RI increased significantly in 4 joints, as indicator of successful treatment. PSV decreased significantly in 3 joints and EDV decreased significantly in 5 joints (paired sample *t* test) (Table 3). The ultrasonographic changes of quantitative parameters in a single patient are presented in Figure 4.

**Table 3:** Quantitative Doppler spectral parameters at baseline and after treatment (mean ± standard deviation)

Joint	Baseline	Six months	*P* (*t* test for sample pairs)
Resistance index			
UC R	0.51 ± 0.10	0.60 ± 0.10	0.002
UC L	0.49 ± 0.08	0.61 ± 0.11	0.001
MCP 3R	0.46 ± 0.09	0.61 ± 0.12	0.003
MCP 5 R	0.49 ± 0.10	0.62 ± 0.10	0.042
Peak systolic velocity in cm/s			
UC R	7.37 ± 3.31	5.92 ± 3.16	0.049
MCP 2 R	9.78 ± 5.82	8.98 ± 5.98	0.040
MCP 4 R	7.75 ± 2.18	6.56 ± 2.12	0.017
End-diastolic velocity in cm/s			
UC R	3.54 ± 1.86	2.26 ± 1.11	0.002
UC L	3.71 ± 1.44	2.28 ± 1.19	0.001
MCP 3 R	3.81 ± 1.58	2.48 ± 1.75	0.033
MCP 3 L	4.65 ± 1.42	3.28 ± 1.85	0.032
MCP 4 R	3.75 ± 1.08	2.65 ± 0.98	0.025

*UC – ulnocarpal; MCP – metacarpophalangeal; R – right; L – left.

In PIP joints, the differences between baseline and after therapy were not significant. These joints are smaller, and on baseline examination no pathologic changes were observed on gray-scale (erosions, synovial thickening, effusions) or CD (vascularization) in 50%-97% of PIP joints. Since in most PIP joints increased vascularization could not be demonstrated initially it was impossible to determine PSV, EDV, and RI. The only PIP joint with a significant difference in EDV was the left PIP III, in which EDV decreased from 5.52 ± 0.12 cm/s to 2.74 ± 0.00 cm/s after therapy (paired sample *t* test, *P* = 0.019).

### Changes of qualitative and quantitative US variables in MCP and UC joints

After therapy, 12 MCP and UC joints showed significant differences in all US qualitative and quantitative variables (Table 4). In 12 joints there was a significant change in at least one parameter. In 6 out of 12 joints, significant changes were observed in 2 or more parameters. In 2 UC joints, changes were observed in 3 or more parameters. Interestingly changes were much more numerous on the joints of the right hand (14 vs 8).

**Table 4:** Distribution of significant changes of qualitative and quantitative ultrasonographic variables after treatment in MCP and UC joints of both hands*

Joint	Synovial thickening	Effusions	Vascularization	RI	PSV	EDV
UC R			↓	↑	↓	↓
UC L	↓			↑		↓
MCP1 R	↓	↓				
MCP1 L	↓					
MCP2 R					↓	
MCP2 L		↓				
MCP3 R	↓		↓	↑		↓
MCP3 L						↓
MCP4 R					↓	↓
MCP4 L	↓					
MCP5 R	↓			↑		
MCP5 L	↓					

*UC – ulnocarpal; MCP – metacarpophalangeal; R – right; L – left; RI – resistance index; PSV – peak systolic velocity; EDV – end-diastolic velocity; ↓ – statistically significant decrease; ↑ – statistically significant increase.

The relation between RI and clinical activity DAS 28 score of >3.2 was investigated by testing the validity of different cut-off RI values for patients before and after therapy. In the “before treatment group,” the best indicators were found for the cut-off value of RI<0.40 with the sensitivity, specificity, positive predictive value, and negative predictive value of 100%. In the “post-treatment group,” the best indicators were found for the cut-off value of RI≥0.55, with the sensitivity of 100%, specificity of 87%, positive predictive value of 94%, and negative predictive value of 100%.

## Discussion

This study confirmed that US of UC and MCP joints was useful in diagnosis and treatment of RA and that quantitative Doppler parameters provided important information about disease activity and treatment efficacy.

It is difficult to predict the course of RA in an individual patient. Most patients have active disease but with considerable intensity fluctuations, resulting in different degree of joint disorder and functional damage (1). Therapy rarely completely inhibits bone erosions, and the absence of the erosion progression is a good indicator of an effective treatment (3). We did not observe significant sonographic changes in the stage of bone erosions after treatment, which indicates that US monitoring of erosions is not clinically relevant, at least in a relatively short six-month follow-up period.

Several studies have shown that gray-scale and CDUS demonstrated inflammatory changes in patients with RA, estimated the extent of the joint disease, and evaluated changes after medical therapy (7-14). Some studies have shown that US can evaluate the effects of tumor necrosis factor alpha-antagonist drugs treatment in patients with RA (12,18-23). Recently it has even been shown that RA patients in clinical remission and with US-defined active synovitis exhibited higher disease activity and increased serum levels of angiogenic biomarkes (24).

Most US studies in RA so far have been performed on small joints of hands and fingers, but examinations are expanding to larger joints as well, like in a study that recently evaluated US score for large joints (shoulder, elbow, hip, knee), the so called SOLAR score (sonography of large joints in rheumatology) (25). Moreover, Ribbens (12) and Terslev (26) noted that Doppler spectral analysis had the potential to estimate inflammation in RA.

The strength of this study is that it evaluated a large number of gray-scale and CDUS features on a large number of hand joints in a highly-standardized fashion, by a single physician. However, the number of patients was small, only thirty, which is the major weakness of the study.

Synovial thickening was significantly reduced after therapy in 7 of 12 MCP and UC joints, and this seems to be the most important morphologic gray-scale feature that should be evaluated in patients with RA, rather than effusions or erosions.

Inflammatory hyperemia is visualized on CDUS as hypervascularization (higher number of small blood vessels with detectable slow flow) and decreased RI on spectral analysis. Decreased RI is the consequence of increased diastolic flow due to reduced resistance and vasodilatation during inflammatory hyperemia. Reduced flow velocity, reduced level of vascularization, elevated RI in treated patients, and correlation between qualitative (vascularization level) and quantitative (RI, PSV, EDV) Doppler parameters and clinical stage of disease (DAS 28) could have major clinical implications. We demonstrated that CDUS features could be analyzed in MCP and UC joints, and that successful treatment results in the reduction of velocities and RI increase. Changes in quantitative Doppler parameters were observed in both UC joints and in 5/10 MCP joints. Our results indicate that RI<0.40 could be a useful threshold value to diagnose clinically active disease, while RI of 0.55 and higher could indicate successful treatment results. However, we used a highly-selected group of patients with pronounced clinical signs of active disease on the initial examination, and cut-off values were determined for patients with moderate disease activity who had DAS 28 score >3.2. Our results are in accordance with those by Terslev et al (26) and Varsamidis et al (27), who demonstrated that hand-joint changes may be quantified with Doppler and that RI increased during therapy.

Qualitative and quantitative Doppler parameters reflect the stage of inflammatory hyperemia and provide the information that cannot be obtained with CR. CR cannot demonstrate or quantify vascularization, but only the changes on joint surfaces that are the consequence of inflammation. On the other hand, scintigraphy and MRI indirectly show inflammatory activity but cannot quantify it. US has considerable advantages to other imaging modalities, since it is noninvasive, does not expose patients to radiation, does not require injection of contrast media, and is widely available and cheap. Utilization of qualitative and quantitative US parameters during treatment could be a convenient noninvasive way to estimate the effectiveness of therapy. CDUS examination should therefore be part of the follow-up algorithm for patients with RA during treatment.

No significant differences between baseline and end of therapy were observed on PIP joints, with only one joint as exception. It is quite hard to visualize vascularization with CD, ie, to demonstrate Doppler signal in these small joints, which is a prerequisite for evaluation of quantitative spectral parameters. Therefore, due to time-consuming and difficult examination, PIP joints can be omitted from studies that evaluate treatment efficacy in RA, which is an important finding of this study.

More significant changes were observed on the right than on the left hand, maybe because the right hand was examined first and as the time passed the examiner got less precise. Further studies are needed to address this issue and investigate if this finding might be related to the fact that the right hand is used more by right-handed people.

For the accurate estimate of the treatment efficacy, the patient has to be examined in a similar fashion before and after therapy. In our study, US examinations were performed in a highly standardized fashion. The quality of image and sensitivity of vascularization detection with Doppler depends on a quality of the scanner, and patients’ findings may differ considerably if they are examined on different scanners or using different gray-scale and Doppler settings on the same scanner. In US studies, the experience of the examiner is crucial. In this study, in order to eliminate or reduce errors all patients were examined before and after treatment on the same high quality state-of-art ultrasound scanner with the same, high quality high-frequency transducer, with similar settings and scanning parameters (gain, electronic focusing, dynamic range) by a single examiner. However, all this is not feasible in daily clinical practice. It requires time, high quality scanners, and maximum concentration of the examiner. An experienced examiner needs some 60 minutes to examine 22 joints of every patient and to perform all the necessary measurements. Our results, however, indicate that 10 PIP joints can be excluded from the examination protocol, which considerably reduces examination time and makes CDUS more feasible in daily clinical practice. This is in accordance with the recent studies by Naredo et al (28), who proposed 12-joint evaluation and Perricione et al (21), who proposed 6-joint evaluation for joint inflammation in RA (wrist, second MCP, and knee), and another study by Naredo et al who proposed wrist, ankle, and MTP joint assessment for detecting residual gray scale and Doppler joint inflammation (29).

US-estimated alterations are partly quantifiable and can be compared to the patient’s clinical state and indicators of disease activity. Contrast-enhanced US may provide even better sensitivity for detection of abnormal vascularization and quantification of changes during treatment. A drawback of this study is that many patients considered to be in clinical remission according to the DAS and ACR/EULAR definitions may still have residual synovitis on US, which makes prognostic significance of US findings debatable (30).

In conclusion, gray scale and CDUS are useful to detect changes on hand joints in patients with RA and to monitor the effectiveness of the therapy. US parameters highly correlate with the laboratory and clinical indicators of disease activity. Reduction of synovial thickening is the best gray scale indicator of treatment efficacy, while Doppler findings that correlate with clinical improvement are decreased vascularization, accompanied by elevation of RI, and decrease in PSV and EDV. The novelty of this research is that it demonstrated the importance of quantitative Doppler parameters and that it found that the cut-off values of RI of 0.40 at baseline and 0.55 after the treatment indicated the presence of active disease and the efficacy of treatment, respectively. We found that only UC and MCP joints should be examined, while PIP joints can be omitted from examination protocol, which is an important finding for the clinical utilization of CDUS in RA.
